# Existence of Lymphatics in Pseudo-memembranes

**Published:** 1843-05

**Authors:** 


*Existence of Lymphatics in pseudo-membranes.*—Mr. Hamilton was shown by Professor Vanderkelk of Utrecht, some preparations showing the existence of lymphatics in new and abnormal parts, such as the effusions and adventitious membranes which inflammation often leaves between the separate layers of serous membranes, and where beyond dispute, they must be newly-formed lymphatics in parts themselves new. "The first preparation," says Mr. H., "placed in my hand was of this nature. The pleura, on its sacral aspect, had been inflamed, had thrown out coagulable lymph, which, by a considerable bridle, connected it with a part of the diaphragm. The surface of this connection was not less than an inch or two in extent, and the bridle varied from a quarter of an inch to an inch in length. The lymphatics of these parts having been injected with quicksilver, the vessels of this system belonging to the lungs and pleura were very conspicuous; not less so those of the diaphragm; and thirdly, not less apparent than either, were distinct beaded lymphatics running along the effused membranes connecting the two normal tissues. As in this portion of the pleura, so it was equally conspicuous in another preparation—a case of lymph effused between the pleura costalis and pulmonalis, the bridle crossing like a regular bridge, and in this evidently adventitious membranous bridge were the lymphatics coursing their way as conspicuously as in the other parts."—*Lond. Med. Gaz.*, Jan. 6, 1843, from *Amer. Journ. Med. Sci.*

---

*Abscess of the Brain.*—In the *Archives Générales de Médecine*, a case of abscess of the right lobe of the brain is published, involving some interesting and important facts. A girl, a servant at a public-house, in a struggle with some drunkards, received a blow from a bottle on the lateral and upper part of the forehead, which caused a large and very contused wound exposing the bone. She was admitted into the hospital on the 9th of December, 1841, under the care of Blandin, and on the 13th of January, after the separation of several sequestra, the wound had cicatrised. The cure, however, was only apparent; a local pain remained, which increased in severity, causing the patient to scream loudly and prevented her moving her head. Vomiting and insomnia followed, but the intellect remained clear, and also all the other faculties. The pulse continued regular, but the patient gradually getting worse, and her general health evidently suffering much, Blandin, *en désespoir de cause*, proceeded to trephine, thinking the irritation might be caused by a sequestrum or a purulent effusion. A piece of the cranium about an inch in diameter was accordingly removed, but no fracture was discovered; the parts beneath, however, presented an equivocal feeling of elasticity to the finger. Under these circumstances, Blandin determined to wait, in the hope that the abscess, if there was one, would open spontaneously, but after the lapse of some time, no such result ensuing, an exploratory puncture was made with care, but without any advantage. For a time, after these operations, the poor girl seemed to be relieved, but the pain soon became more severe, and emaciation and prostration, followed by a severe attack of erysipelas, ushered in death. The opening made in the cranium by the trephine had not closed, and the dura mater was intact. On passing in the finger after death, the same elastic feeling was perceived as during life, and on examination, it was found to correspond to a large circumscribed encysted abscess in the right lobe, separated from the dura mater by a thick layer of cerebral substance. It is clear that if Blandin had incised this layer, the abscess would have been discharged, and the patient had a chance for life.

Aran, the reporter of the case, in his remarks upon it, draws attention to the great neglect with which the trephine is now treated, and although far from an advocate for its general use, he equally condemns its total abandonment; he also thinks that in serious cases, such as that under notice, the surgeon not deriving benefit from the treatment, should not hesitate to plunge a narrow bladed bistoury into the brain itself, and to some depth if required.—*Prov. Med. Journ.*, Dec. 17, 1842.—*Amer. Journal.*

